# They Are What You Hear in Media Reports: The Racial Stereotypes toward Uyghurs Activated by Media

**DOI:** 10.3389/fnins.2017.00675

**Published:** 2017-12-06

**Authors:** Jia Jin, Guanxiong Pei, Qingguo Ma

**Affiliations:** ^1^Business School, Ningbo University, Ningbo, China; ^2^Academy of Neuroeconomics and Neuromanagement, Ningbo University, Ningbo, China; ^3^School of Management, Zhejiang University, Hangzhou, China; ^4^Institute of Neural Management Sciences, Zhejiang University of Technology, Hangzhou, China

**Keywords:** stereotypes, ERPs, Uyghurs, N400, Han Chinese, media

## Abstract

Stereotypes from the major nationality toward minorities constitute a widely concerning problem in many countries. As reported by previous studies, stereotypes can be activated by media information that portrays the negative aspects of the target group. The current study focused on the neural basis of the modulation of negative media information on Han Chinese stereotypes toward Uyghurs by using event-related potentials. We employed the lexical decision task, in which participants were asked to categorize the presented word as positive or negative. Behavioral result showed that participants had a shorter reaction time to positive adjectives than to negative adjectives. The data of brain activity showed that compared with the Han condition, the Uyghurs condition elicited smaller N400 differences in the media priming group, whereas there was no significant N400 deflection difference between Han Chinese and Uyghurs in the control group. The current results suggested that the negative media information might influence their judgments toward other groups reflected in the deflection of N400 amplitude. Therefore, in order to mitigate or even eliminate stereotypes about national minorities, the effort of the media is important.

## Introduction

Individuals' negative stereotypes toward outgroup individuals may lead to social problems, such as race bias between nationalities, which has been commonly reported by previous studies. For instance, Whites' prejudice toward Blacks has been widely investigated by researchers (e.g., Crosby et al., 1980). Dovidio and colleagues conducted an experiment to study Whites' stereotypes toward Blacks by investigating the association between schematic faces of Black/White and positive/negative words. Their results showed that the White participants responded faster to positive words but slower to negative words following a White prime vs. following a Black prime. The authors explained it as the result of Whites' stereotypes toward Blacks (Dovidio et al., 1997).

Similar to America, China is a multinational country. As Han people make up 93% of the Chinese population, their attitude toward other national minorities is of great importance for social stability. Thus, many researchers focused on the racial prejudice phenomenon in China, especially the images and social distance among Han Chinese, minorities and foreign nationalities. For example, Fong and Spickard (1994) studied ethnic relations in China. Their results showed that Han Chinese felt affinity for overseas Chinese and Uyghurs but felt extreme distance from Tibetans (Fong and Spickard, 1994). Yee (2003) conducted fieldwork in Urumqi, the provincial capital of Xinjiang Uyghur Autonomous Region, to study ethnic relations and found there was a low degree of integration between the two ethnic groups (Yee, 2003). These studies showed the existence of negative stereotype between nationalities in China. It is natural to ask if there are any ways to change or eliminate the negative stereotype.

Based on the social category theory, stereotypes derived from category-based responses to other people on the basis of social distinctions such as race, gender, and age (Bodenhausen and Macrae, 1998; Fiske et al., 1999). Therefore, people are motivated to overcome the stereotypes by avoiding responding to targets primarily on the basis of a category (Fiske and Neuberg, 1990; Plant and Devine, 1998). Categorization happens often based on the visually prominent and culturally relevant features (Brewer and Feinstein, 1999). As culturally relevant features are hard to be changed, the prominent visual information is always adapted to change or eliminate the stereotype. It was suggested that exposure to stereotype-associated stimuli may activate the stereotype. This may be applied generally to the perceptions and evaluations of outgroup others (Bargh et al., 1996; Lepore and Brown, 1997). Specifically, the available stereotypic information tends to lead to stereotype application in subsequent judgments of stereotyped group members (Henderson-King and Nisbett, 1996).

Previous studies have shown that media, which is a main source of external information, played an important role in stereotype activation. For instance, researchers have suggested that depictions of African Americans as criminal, aggressive, and unintelligent in the media help reinforce and maintain hostile anti-black prejudice against this African Americans (Oliver, 1999; Dixon and Linz, 2000). Similarly, researchers also found that when the Western media portrayed third-world people as naive, inferior, traditional, and uncivilized, it rationalized the perpetuation of benevolent, paternalistic prejudice toward these people (Mitra, 1999; Ramasubramanian, 2005). There were also studies which tried to employ the media to reduce intergroup prejudice (Ramasubramanian, 2007; Paluck, 2009). Therefore, in the current study, we intend to investigate whether there are also racial biases and stereotypes in China and if these biases and stereotypes will be impacted by the media. Specifically, we focus on Han's attitude toward Uyghurs, which is one of the main national minorities in China, and how media information influences this effect.

In comparison to the descriptive research on media stereotypes, relatively few studies had investigated the cognitive processes involved in media stereotyping. Some sociologists have applied research techniques from cognitive neuroscience, such as eye-tracking, functional magnetic resonance imaging (fMRI) and event-related potentials (ERPs), to investigate the cognitive processes of racial prejudice and stereotyping in recent years (Payne, 2001; Amodio et al., 2004; Correll et al., 2006). For example, Wang et al. (2011) investigated the stereotypes about rural migrant workers (RMW) in China by using event-related potentials and found the RMW-positive adjective condition elicited larger N400 amplitude than the urban worker-positive adjective condition, which revealed the negative stereotypes about RMWs (Wang et al., 2011). Therefore, in the current study, we also intend to employ ERPs to investigate the cognitive processes of media stereotyping of Han Chinese toward Uyghurs on brain level.

Based on previous ERPs studies about stereotyping (White et al., 2009; Hehman et al., 2014), the N400 is considered as the index of stereotypes. The N400 component was first studied by Kutas and Hillyard (1980) by using an anomalous sentence task. They found that the N400 was a negative ERP deflection that appeared when there was semantic incongruity of a sentence and typically peaked around 400 ms at central-parietal electrode sites (Kutas and Hillyard, 1980). In subsequent studies, researchers also found that the N400 could be elicited by semantic incongruity of word pairs (Franklin et al., 2007). That is, when the second word had no semantic relationship with the first word, it elicited larger N400 amplitude. Additionally, researchers have suggested that a stereotype can also be defined as a specific class of semantic association sorted by memory (Amodio et al., 2008; White et al., 2009). Therefore, a lexical decision task is often used to study stereotypes and racial prejudice, and the N400 component is considered as a neural index. For example, in a study by White et al. (2009), participants were primed by a gender category (Women or Men) followed by a word that was either consistent or inconsistent with a gender stereotype. The stereotype-incongruent word pairs elicited larger N400 amplitudes compared to the stereotype-congruent word pairs (White et al., 2009).

Based on the aforementioned literatures, we believed that semantic mismatch in the lexical decision task would affect the amplitude of the N400 and possibly reveal a stereotype effect. Specifically, the incongruity between the meaning of the second stimuli and the first stimuli would elicit larger N400 amplitude than the congruent condition. On the other hand, according to previous studies about media effect of stereotype, we hypothesized that Han's stereotypes toward Uyghurs would be enhanced by negative media information about Uyghurs. Thus, we expected that the incongruity between Uyghurs and positive adjectives would be larger and/or the congruity between Uyghurs and negative adjectives would be smaller in the media priming group. That is, compared with the control group, the N400 amplitude induced by negative adjectives would be smaller and/or the N400 amplitude induced by positive adjectives would be larger under the Uyghurs condition in the negative media priming group. This phenomenon may not be found under the Han condition since no negative media information about Han was provided. In order to state the results more clearly, we intend to extract the N400 difference wave by minus the positive adjectives condition from the negative adjectives condition. Therefore, smaller d-N400 means participants considered the previous shown photo more incongruent with positive adjectives and/or more congruent with negative adjectives. This suggested the larger degree of negative stereotypes toward Uyghurs. Therefore, we hypothesized the d-N400 between negative adjectives and positive adjectives following Han Chinese would be larger than those following Uyghurs after priming with negative media information. The hypotheses are also summarized in the following Table 1.

**Table 1:** Summary of hypotheses for N400 and d-N400 amplitudes.

**Group**	**Stereotype**	**Hypotheses for N400 amplitude**	**Hypotheses for d-N400 amplitude**
Control	No difference between two nationalities	Negative adjective> Positive adjective for both nationalities	No significant difference for d-N400 amplitude of the two nationalities
Priming	Stereotype was activated only in Uyghur condition but not in Han Chinese condition	Han condition: negative adjective>positive adjective, almost the same as control group	Han >Uyghur
		Uyghur condition: negative adjective induced similar or even smaller N400 than positive adjective	

## Methods

### Participants

In all, 36 graduate or undergraduate students (18 male) were recruited from Zhejiang University, ranging in age from 20 to 27 years old (mean age = 23.03; SD = 1.70) to participant the EEG experiment. Another 50 graduate or undergraduate students (25 male) were recruited, ranging in age from 20 to 27 years old (mean age = 23.03; SD = 1.70) to participant in the same experiment behaviorally without recording their EEG data. They were all of Han nationality. Their mother tongue was Mandarin Chinese. They self-reported right-handedness. Participants had normal or corrected-to-normal vision, and they were screened for a history of neurological and psychiatric disorders, substance abuse, and current psychotropic medications. The participants were assigned randomly to two groups (priming vs. control) before the event-related potentials (ERPs) experiment. For the EEG experiment, in each group, there were 18 participants (9 males). For the behavioral experiment, there were 43 participants (22 males) in each group. Informed consent was obtained from all participants, and the research was approved by the Ethical Committee of the Neuromanagement Lab of Zhejiang University. All the participants claimed to have unprejudiced attitudes toward the Uyghur nationality, and they were paid 35 RMB for participation. The data from one subject in the priming group were discarded for excessive recording artifacts, and the data from one subject in the control group were discarded for misunderstanding the rules of the categorization task, resulting in low accuracy (56.67%). Thus, 34 valid subjects were included in the final EEG data analysis and 84 valid subjects were included in the final behavioral data analysis.

### Stimuli and procedures

The stimuli consisted of 240 image-word pairs (12 facial images × 20 adjectives) divided randomly into 4 blocks. We obtained facial images from graduates (with their permission to be used in this study) of the Xinjiang University, which is located in the Xinjiang Uyghur Autonomous Region. Six facial images were of individuals of Uyghur nationality (half male and half female), and another six were of individuals of Han nationality (half male and half female). The images were unfamiliar to the participants (no schoolmates or celebrities) and were edited by Adobe Photoshop 10.0 (Adobe Systems Incorporated, San Jose, California, USA) to a uniform size (4.23 cm by 6 cm, 240 by 340 pixels). After the experiment, participants were asked to classify the 12 facial images into two groups, the “Uyghur nationality” group or the “Han nationality” group. All of them gave the correct answer. The 20 adjectives (half positive and half negative) were derived from Wang et al. (2011)and are shown in Table 2. The 240 image-adjective pairs were divided into four conditions: Uyghur nationality-positive adjective, Uyghur nationality-negative adjective, Han nationality-positive adjective, and Han nationality-negative adjective.

**Table 2:** Selected Chinese adjectives translated into English.

**Positive adjectives**	**Negative adjectives**
confident, righteous, civilized, polite, elegant, wise, noble, gentle, decent, and dignified	dirty, clumsy, shortsighted, rude, lazy, mean, extreme, narrow-minded, stingy, and barbaric

## Procedure

Participants were comfortably seated in a dim, sound-attenuated and electrically shielded room. The instructions for the experiment were presented on written paper. The experimental stimuli were presented in the center of a computer-controlled CRT monitor at a distance of 100 cm (with a visual angle of 2.42° × 3.44°). All the stimuli, recording triggers, and behavioral responses were presented and recorded with the E-Prime 2.0 software package (Psychology Software Tools, Pittsburgh, Pennsylvania, USA). A keypad was provided to the participants to make choices. Participants were informed that they would see a series of image-word pairs and their task would be to categorize the word as positive or negative as quickly as possible. The experiment included four blocks for both the priming and control group with each block included 60 trials.

**Figure 1:**
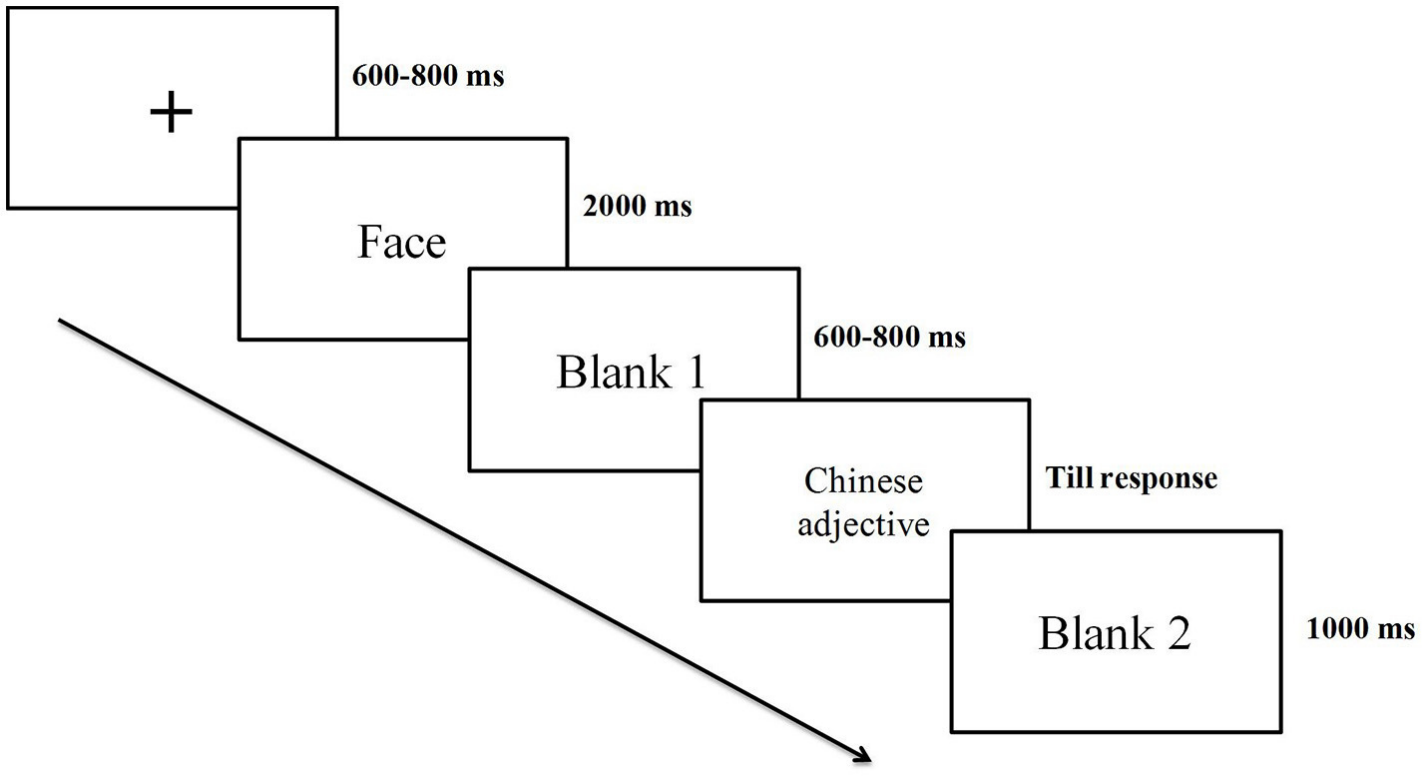
Experimental task: Participants were instructed to categorize the word as positive or negative. EEGs were recorded from the subjects throughout the experiment.

A single trial is shown in Figure 1. In each trial, a central fixation cross was presented for a duration varying randomly between 600 and 800 ms. Then, the facial image was presented at the center of the screen for 2,000 ms against a white background. After a random blank screen that lasted for 600–800 ms, the Chinese adjective was shown. The participant pressed one of two keys to indicate their classification. The response key corresponding to positivity or negativity was counterbalanced across participants. The trials were presented randomly, and the interval across trials was 1,000 ms.

Prior to the initiation of the formal experiment, each participant practiced five trials to familiarize themselves with the procedure. More importantly, there was a one-minute-long video show before the formal experiment for the priming group but not for the control group. The video was derived from a television program about ethnic clashes between the Uyghur nationality and Han nationality in Urumqi on July 5th, 2009. It reported there was a series of violent riots in Urumqi, which began as a protest of Uyghurs but escalated into violent attacks that mainly targeted Han people. Some Hans died. Many vehicles and buildings destroyed.

### EEG recording

The EEG was recorded (band-pass 0.05–70 Hz, sampling rate 500 Hz) with a Neuroscan Synamp2 Amplifier (Scan 4.3.1, Neurosoft Labs, Incorporated, Sterling, Virginia, USA) using an electrode elastic cap (64 Ag/AgCl electrodes according to the standard international 10–20 system). A frontal electrode site between FPz and Fz was used as the ground. There were two mastoid electrodes, and the left was used as a reference. Horizontal and vertical electrooculograms (EOG) were monitored with two pairs of electrodes, one pair located below and above the left eye in parallel with the pupil, and the other pair situated 10 mm from the lateral canthi. The experiment started only when the electrode impedances were kept under 5 kΩ.

### EEG/ERP data analyses

The EEG data were analyzed using Neuroscan 4.3.1. Data were re-referenced to the algebraically computed average of the left and right mastoids for further analysis. The EOG artifacts with ocular movements were corrected using the algorithm proposed by Semlitsch et al. (1986). The EEGs were digitally low-pass filtered at 30 Hz (24 dB/Octave) and were segmented into epochs from 200 ms before stimulus onset to 1,000 ms after stimulus onset. Baseline correction was performed with the first 200 ms of each channel. Trials that contained amplifier clipping, bursts of electromyography activity, or peak-to-peak deflections that exceeded ±80 μV were excluded from the final average. More than 40 sweeps for each condition remained. During facial image presentation, the EEG epochs were averaged for the ethnic Han's face and the ethnic Uyghur's face. During the Chinese adjective presentation, the EEG epochs were separately averaged for nationality (Han/ Uyghur) and adjective (positive/negative).

## Statistical analysis

For the behavioral data analysis, the response accuracy and the reaction times for correct responses to adjectives were separately analyzed with a 2 (nationality: Han vs. Uyghur) × 2 (adjective: positive vs. negative) × 2 (group: priming vs. control) mixed-design repeated-measures ANOVA.

In further EEG analysis, we chose the time window from 320 to 430 ms after the adjective onset to analyze the mean amplitude of the N400 on the basis of the visual observation of the grand average waveforms and on the guidelines provided by Picton et al. (2000). We selected nine electrode sites for the statistical analysis: F1/z/2, C1/z/2, and P1/z/2 in frontal, central and parietal areas. We carried out a 2 (nationality: Han vs. Uyghur) × 2 (group: priming vs. control) × 2 (adjective: positive vs. negative) × 9 (electrode: F1/z/2, C1/z/2, and P1/z/2) mixed-design repeated-measures ANOVA for N400 analysis. Similarly, a 2 (nationality: Han vs. Uyghur) × 2 (group: priming vs. control) × 9 (electrode: F1/z/2, C1/z/2, and P1/z/2) mixed-design repeated-measures ANOVA for differentiated N400 analysis (d-N400: negative/positive N400 discrepancy, which reflects the degree of bias) was also conducted. A simple-effect analysis was carried out when any significant interaction effect among factors appeared. The Greenhouse–Geisser correction was applied in all statistical analyses when necessary (Greenhouse and Geisser, 1959).

## Results

### Behavioral results

For the 2 (nationality) × 2 (adjective) × 2 (group) mixed-design ANOVA of response accuracy, no significant main effect or interaction effect for these factors was found (*ps* > 0.05). For the 2 (nationality) × 2 (adjective) × 2 (group) mixed-design ANOVA of reaction time, the results showed a significant main effect of adjective [*F*_(1, 82)_ = 19.181; *p* < 0.01; η^2^ = 0.190], which indicated that participants had a shorter reaction time toward positive adjectives (*M* = 753.419 ms, S.E. = 26.264) than toward negative adjectives (*M* = 784.982 ms, S.E. = 26.689). The main effect of nationality was marginally significant [*F*_(1, 82)_ = 3.770; *p* = 0.056; η^2^ = 0.044], and the reaction time toward the Uyghur nationality (*M* = 777.049 ms, S.E. = 29.030) was longer than the Han nationality (*M* = 761.352 ms, S.E. = 23.793). However, no significant main effect was found by group (*p* > 0.05). There was also no interaction effect among these factors (*ps* > 0.05). All the results were summarized in Table 3.

**Table 3:** F statistics and *p*-value of behavioral results.

**Source**	**Response accuracy**		**Reaction time**	
	***F***	***p*-value**	***F***	***p*-value**
nationality	2.785	0.099	3.770	0.056
adjective	0.872	0.353	19.181	<0.001
group	0.079	0.780	0.663	0.418
nationality* adjective	1.604	0.209	0.283	0.596
nationality* group	0.988	0.323	1.269	0.263
adjective* group	0.723	0.398	3.274	0.074
nationality* adjective* group	0.114	0.737	2.680	0.105

### ERP results

#### N400 analysis

For the 2 (nationality) × 2 (group) × 2 (adjective) × 9 (electrode) mixed-design ANOVA of N400, the results showed no significant main effects (*ps* > 0.1) for nationality and group. The interaction effect of nationality and group, nationality and adjective as well as adjective and group were not significant (*ps* > 0.1). However, the main effect of adjective [*F*_(1, 32)_ = 52.279; *p* < 0.001; η^2^ = 0.620] as well as the interaction effect of nationality, adjective and group was notable [*F*_(1, 32)_ = 5.178; *p* = 0.030; η^2^ = 0.139].

Therefore, simple-effect analyses were conducted. In the priming group, the main effect of nationality was not significant (*p* > 0.1). The main effect of adjective [*F*_(1, 16)_ = 26.017; *p* < 0.001; η^2^ = 0.619] and interaction effect of nationality and adjective [*F*_(1, 16)_ = 13.127; *p* = 0.002; η^2^ = 0.451] were notable. Further simple-effect analyses were also conducted. For the Han nationality, the negative adjective (*M* = −1.147 μV, SE = 0.536) induced larger N400 amplitude than that of positive adjective (*M* = 1.018, μV, SE = 0.609). However, there was no significant N400 difference for negative and positive adjective under the Uyghur condition. In the control group, only the main effect of adjective was significant [*F*_(1, 16)_ = 27.008; *p* < 0.001; η^2^ = 0.628]. The main effects of nationality as well as the interaction effect of nationality and adjective were not significant (*ps* > 0.1).

#### N400 difference wave analysis

**Figure 2:**
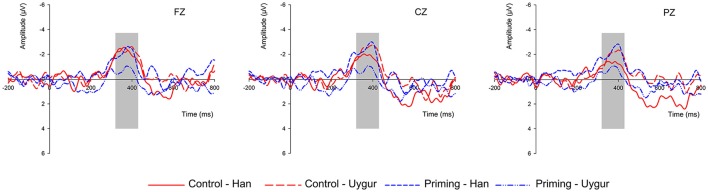
Grand-averaged ERP waveforms in the whole brain regions with three electrodes: Fz, Cz, and Pz. N400 difference comparison between the two groups (media priming group vs. control group) and two nationalities (Han vs. Uyghur). The time window for the d-N400 was 320–430 ms.

In order to descript the results more clearly, the N400 difference wave was also analyzed. For the 2 (nationality) × 2 (group) × 9 (electrode) mixed-design ANOVA of d-N400, the results showed no significant main effects (*ps* > 0.05). However, the interaction effect of nationality and group was significant [*F*_(1, 32)_ = 5.178; *p* = 0.030; η^2^ = 0.139]. Further simple-effect analysis indicated that, in the priming group [*F*_(1, 16)_ = 13.127; *p* = 0.002; η^2^ = 0.451], the Uyghur nationality (*M* = −0.655 μV, SE = 0.328) induced a significantly less negative d-N400 than that of the Han nationality (*M* = −2.165 μV, SE = 0.364), but there were no significant d-N400 differences between the Uyghur nationality (*M* = −2.023 μV, SE = 0.551) and the Han nationality (*M* = −1.671 μV, SE = 0.448) in the control group (*p* > 0.1). When only the Uyghur nationality was analyzed, the effect of group was significant [*F*_(1, 32)_ = 4.545; *p* = 0.041; η^2^ = 0.124], and the mean d-N400 in the priming group (*M* = −0.734 μV, SE = 0.303) was less negative than that in the control group (*M* = −1.504 μV, SE = 0.611). There were no significant d-N400 differences between the priming group (*M* = −1.958 μV, SE = 0.360) and the control group (*M* = −1.707 μV, SE = 0.402) when only the Han nationality was analyzed (*p* > 0.1), as shown in Figure 2.All the results of N400 amplitude and d-N400 in the different conditions are summarized in Table 4.

**Table 4:** Amplitudes and statistic results of N400 and N400 difference wave.

**Group**	**Experiment condition**	**N400 amplitude (μV)**	**N400 difference amplitude(μV)**	**Simple-effect analysis pair**	**Result**
Control	Han negative (HN)	−0.598	−1.671	Control: HN vs. HP for N400	N/A
	Han positive (HP)	1.103		Control: UN vs. UP for N400	N/A
	Uyghur negative (UN)	−1.126	−2.023	Priming: HN vs. HP for N400	Negative> Positive
	Uyghur positive (UP)	0.897		Priming: UN vs. UP for N400	Not significant
Priming	Han negative (HN)	−1.147	−2.165	Control: Han vs. Uyghur for d-N400	Not significant
	Han positive (HP)	1.018		Priming: Han vs. Uyghur for d-N400	Han > Uyghur
	Uyghur negative (UN)	−0.390	−0.655	Han: Control vs. Priming for d-N400	Not significant
	Uyghur positive (UP)	0.265		Uyghur:Control vs. Priming for d-N400	Control> Priming

## Discussion

Using ERP measurements, the present study performed a brain-level analysis of the mechanism underlying modulation of negative media reports on Han Chinese stereotypes toward a national minority, the Uyghurs. A lexical decision task was conducted in two groups, control group and media priming group.

Behavioral results showed that participants had a shorter reaction time to positive adjectives than to negative adjectives. Previous studies about stereotypes have suggested that the accessible attitudes are faster at recognizing stereotypical words. As a result, the incongruent condition would have longer reaction time than the congruent condition (Fazio et al., 1995; Dovidio et al., 1997). Therefore, the current result revealed that for the impression of both Uyghurs and Han Chinese, positive adjectives are more congruent.

At the brain level, we found that the N400 difference induced by negative adjectives and positive adjectives was similar between two nationalities in the control group. However, in the media priming group, the Uyghur nationality induced a significantly less negative d-N400 than the Han nationality. That is, compared with the Han nationality, the N400 difference induced by the negative adjective and the positive adjective was smaller toward the Uyghur nationality. More specifically, after the negative priming from media information, the N400 amplitude induced by negative adjectives became smaller and/or the N400 amplitude induced by positive adjectives became larger under the Uyghurs condition compared with the control group. These results are in accordance with our hypotheses which are summarized in Table 1. According to the above-mentioned literatures on the N400, the N400 deflection can be considered an index of stereotypes (Rasmussen, 2007; White, 2008; Hehman et al., 2014). Therefore, the current results suggest that negative stereotypes only occurred when the participants had been primed with negative information about Uyghurs. This was consistent with previous studies which stated that the related negative media information could increase the stereotype effect. For example, an early study on gender stereotypes showed that stereotypic rock music videos increased the accessibility of sex role stereotypic schemas (Hansen and Hansen, 1988). This study supported the notion that the media information could enhance the gender stereotype effect. Furthermore, some studies also showed that media information could also influence stereotype effect toward another nationality. Such as, a study conducted by Givens and Monahan examined how mediated portrayals of African American women influenced judgments of them. Their results showed that when evaluating a job interviewee, the jezebel stereotype video increased participants' negative perception toward African American females (Givens and Monahan, 2005).

Researchers also tried to explain this phenomenon theoretically. The model of stereotype activation suggested by Devine and Montiethstated that certain circumstances influence the stereotype effect even though the stereotypes are always activated unintentionally and unconsciously (Devine and Monteith, 2009). That is, without conscious efforts to rectify them, frequent and recent exposure to stereotypes in the environment will more easily influence perceivers' social judgments and impressions about the racial group (Wittenbrink et al., 2001). As what we stated in the introduction part, the category response is related to existing stereotypes. The racial categorization will be enhanced by available visual information (the priming video, in this case) since the presence of priming information facilitates responses to target categories that share meaning, valence, or some other key attribute (Neely, 1977). The associations between race categories and certain attributes are believed to be largely automatic (Fiske, 1998), learned through first-hand experiences with people of the stereotyped groups and second-hand external information, such as the media (Ramasubramanian, 2007). When there is lack of first-hand experiences, the second-hand external information becomes an important consideration for the participants' attitudes toward another group.

In the current study, the participants are Han Chinese from the Zhejiang province, which is far from the Uyghur's community, Urumqi. They have little or even no direct contact with Uyghurs. As a result, their perception of Uyghurs mainly comes from vicarious contact through the media. Thus, the biased information from the media inevitably becomes incorporated into “common knowledge” or schemata that viewers form about Uyghurs. In the media priming group, the participants were exposure in negative media information of Uyghurs, their negative stereotype toward Uyghurs was enhanced, resulting in smaller N400 difference between negative adjective and the positive adjective. These results add to the findings of a number of related studies (Amodio et al., 2004; Conrey et al., 2005) by clarifying one manner in which available information can activate category responses.

However, there are also some limitations in the current study. Firstly, the current study only focused on the negative aspect of media information. Future research can also focus on the positive aspect of media information and develop strategies to mitigate or even eliminate the stereotypes of biased media about national minorities. Secondly, the participants of the current studies are university students, who have little first-hand experiences with Uyghurs. Future studies can focus on the participants who are more familiar with the Uyghurs and investigate if their stereotype would also be activated by negative media information. Thirdly, using one video for induction purposes may not be reliable enough. In the future, researchers can consider using at least 2 or 3 examples of stimuli for such purposes and check that the effect is similar for all of them. Last but not the least, the current study only concerned about the negative media portrayals influences the perception of minorities, but ignored the other social groups. Future studies can investigate this effect by adding a third condition in which the same negative induction scenario is addressed toward the other individuals.

In summary, the current study investigated the associated underlying neural mechanisms of Han Chinese media stereotype toward a national minority, the Uyghurs. The behavioral results showed that participants have a longer reaction time under the positive adjective condition compared to the negative adjective condition regardless of the nationality presented. The ERPs results indicated that compared with Han people, the Uyghurs elicited smaller N400 difference in the media priming group, but this effect was not found in the control group. As N400 amplitude can be modulated by stereotype effect, the current results suggested that when Han Chinese were primed by negative information about a specific group from the media, their negative stereotypes toward people from the target group were activated. That is, the media can influence people's judgments toward other groups in subtle, subconscious ways.

## Author contributions

JJ made substantial contributions to the conception of the work, analysis, and interpretation of data, as well as drafting the manuscript. GP made substantial contributions to the conception of the work, as well as data acquisition, data interpretation. QM made substantial contributions to the conception of the work, as well as the analysis and interpretation of data. All authors gave approval of the final version.

## Ethics statement

This study was carried out in accordance with the recommendations of the Ethical Committee of the Neuromanagement Lab of Zhejiang University with written informed consent from all subjects. All subjects gave written informed consent in accordance with the Declaration of Helsinki. The protocol was approved by the Ethical Committee of the Neuromanagement Lab of Zhejiang University.

### Conflict of interest statement

The authors declare that the research was conducted in the absence of any commercial or financial relationships that could be construed as a potential conflict of interest.
