# Corrigendum: They Are What You Hear in Media Reports: The Racial Stereotypes toward Uyghurs Activated by Media

**DOI:** 10.3389/fnins.2019.00168

**Published:** 2019-03-05

**Authors:** Jia Jin, Guanxiong Pei, Qingguo Ma

**Affiliations:** ^1^Business School, Ningbo University, Ningbo, China; ^2^Academy of Neuroeconomics and Neuromanagement, Ningbo University, Ningbo, China; ^3^School of Management, Zhejiang University, Hangzhou, China; ^4^Institute of Neural Management Sciences, Zhejiang University of Technology, Hangzhou, China

**Keywords:** stereotypes, ERPs, Uyghurs, N400, Han Chinese, media

There is an error in the Funding statement. The correct funding number for the “National Project” is “AWS14J011”.

The authors apologize for this error and state that this does not change the scientific conclusions of the article in any way. The original article has been updated.

